# Ultra-Abrupt Tapered Fiber Mach-Zehnder Interferometer Sensors

**DOI:** 10.3390/s110605729

**Published:** 2011-05-27

**Authors:** Benye Li, Lan Jiang, Sumei Wang, Lanying Zhou, Hai Xiao, Hai-Lung Tsai

**Affiliations:** 1 School of Mechanical Engineering, Beijing Institute of Technology, Beijing, 100081, China; E-Mails: libenye@bit.edu.cn (B.L.); wangsumei@bit.edu.cn (S.W.); zhouly@bit.edu.cn (L.Z.); 2 Department of Electrical and Computer Engineering, Missouri University of Science and Technology, Rolla, MO 65409, USA; E-Mail: xiaoha@mst.edu (H.X.); 3 Department of Mechanical and Aerospace Engineering, Missouri University of Science and Technology, Rolla, MO 65409, USA; E-Mail: tsai@mst.edu (H.-L.T.)

**Keywords:** fiber sensors, refractive index, Mach-Zehnder interferometer, zeolite coating

## Abstract

A fiber inline Mach-Zehnder interferometer (MZI) consisting of ultra-abrupt fiber tapers was fabricated through a new fusion-splicing method. By fusion-splicing, the taper diameter-length ratio is around 1:1, which is much greater than those (1:10) made by stretching. The proposed fabrication method is very low cost, 1/20–1/50 of those of LPFG pair MZI sensors. The fabricated MZIs are applied to measure refractive index, temperature and rotation angle changes. The temperature sensitivity of the MZI at a length of 30 mm is 0.061 nm/°C from 30–350 °C. The proposed MZI is also used to measure rotation angles ranging from 0° to 0.55°; the sensitivity is 54.98 nm/°. The refractive index sensitivity is improved by 3–5 fold by fabricating an inline micro–trench on the fiber cladding using a femtosecond laser. Acetone vapor of 50 ppm in N_2_ is tested by the MZI sensor coated with MFI–type zeolite thin film. The proposed MZI sensors are capable of *in situ* detection in many areas of interest such as environmental management, industrial process control, and public health.

## 1. Introduction

Optical fiber sensors have attracted tremendous research interest in recent years [1–4]. Among many promising photonic devices, fiber-based Mach-Zehnder interferometers (MZI) stand out for their compactness, capability of responding to a wide variety of measurands, and relatively simple fabrication process. A number of fabrication techniques have been proposed to make fiber MZIs, including fiber tapering [5,6], core mismatch [7], imbedded micro air–cavity in fiber [8,9], pairing of LPFGs [10], laser heating induced microbending [11], and microstructure collapsing on a photonic crystal fiber [12].

High-order cladding modes are excited in fiber MZIs, which are guided by the cladding-ambient interface and directly exposed to the environment. The refractive index change of the environment can significantly change the effective propagation constant of the cladding modes. Thus, fiber MZIs can be used as a refractive index sensor by testing the phase shift of interference fringe. Fiber MZIs are also used to detect temperature, curvature, strain and stress [5,7,12]. It was discovered that the unique combination of optical and chemical properties of zeolites could be used to develop optical chemical sensors with high sensitivity [13]. Pure silica MFI-type zeolite thin film on long period fiber gratings was successfully synthesized for highly sensitive detection of organic vapors [14].

Recent developments in femtosecond laser technology have opened new possibilities for one-step fabrication (without additional assembly) of micro/nano-scale three-dimensional (3D) structures in various solid materials with greatly reduced collateral damage. Especially, new opportunities for laser micromachining of fiber optics devices are possible using ultrashort laser pulses. By using femtosecond lasers, various structures have been fabricated in fibers and used in the sensing field [15–18].

Among the various types of MZIs, fiber taper-based structures have the advantages of simplicity and high repeatability [5,6]. To obtain effective coupling between core and cladding modes, large taper angles are preferred [19]. Fiber tapers are usually made by stretching a fiber under high heat or electrical arc conditions, which limits the shortest length of the device. The tapered region has a typical length of ∼600 μm [6]. This study proposes an MZI consisting of two ultra-abrupt fiber tapers fabricated by a fusion splicer using ordinary single mode fibers. By fusion-splicing, the taper diameter-length ratio is 1:1, which is much greater than those (1:10) made by stretching reported in the previous works [5,6]. When it is used as a RI sensor, the sensitivity is comparable to LPFG pair MZI sensors [10] and the taper-based MZI sensors fabricated by stretching method [5,6]. However, the fabrication process is much simpler and has much higher robustness. The refractive index sensitivity is improved by 3–5 fold through imbedding an inline side-ablated micro-trench on the fiber cladding using a femtosecond laser. The MZI sensors are applied for direct measurement of acetone vapor of 50 ppm in N_2_ after coated with zeolite thin film. In addition, temperature and rotation angle tests are also conducted.

## 2. Device Principle

**Figure 1.**
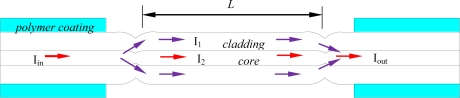
Schematic diagram of the MZI consisting of two ultra-abrupt fiber tapers.

Figure 1 shows the schematic diagram of the MZI consisting of two ultra-abrupt fiber tapers. The input testing laser beam (I_in_) is split into two portions in the first ultra-abrupt taper: one (I_1_) through the core and the other (I_2_) through the cladding. Part of the light (I_1_) in cladding modes is coupled back into the core at the second ultra-abrupt taper. Interference between the light traveling in the cladding and that traveling in the core is detected by measuring I_out_.

The interference signal reaches minimum when the phase difference between cladding and core modes satisfies the following condition [7]:

(1) $$2\pi \;\left(n_{\mathrm{core}}^{\mathrm{eff}}\;-\;n_{\mathrm{cl}}^{\mathrm{eff}}\right)\frac{L}{\lambda_{v}}\;=\;(2k\;+\;1)\pi$$

where *L* is the interferometer length; *k* is an integer; *λ_v_* is the maximum attenuation wavelength of the *k*^th^ order; $n_{\mathrm{core}}^{\mathrm{eff}}$ and $n_{\mathrm{cl}}^{\mathrm{eff}}$ are the effective refractive indices of the core and cladding, respectively.

## 3. Device Fabrication

**Figure 2.**
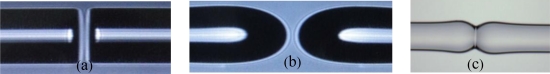
The ultra-abrupt fiber taper fabricated by fusion-splicing. The CCD images of: **(a)** the two normal cleaved fiber ends, **(b)** the two ellipsoidal heads formed by one time discharge; **(c)** The microscopic image of the formed fiber taper.

The fiber inline Mach-Zehnder interferometer is fabricated by the following steps using a conventional fusion splicer (model IFS-9, INNO INSTRUMENT, Inc., Korea): (a) the two normal cleaved ends are made ellipsoidal by a one time discharge (Figure 2(a,b) are the CCD images of the normal cleaved ends and ellipsoidal heads, respectively); (b) the two ellipsoidal heads are moved into contact in the center of the splicer electrodes, and will be fused together by another discharge; (c) the other ultra-abrupt fiber taper is formed through the same process separated by a distance *L*.

Figure 2(c) shows the microscope image of the ultra–abrupt fiber taper after the two ellipsoidal heads are fused together. To minimize the losses and achieve robust splices, the arc duration and current are set 60% (1,200 ms) and 50% of the default value, respectively. The fiber core and cladding are melted together at the ellipsoidal region, and the two ends are touching each other with deformation. Due to the fusion and deformation of the fiber core and cladding, part of the light spread into the cladding at the first ultra-abrupt taper and meet with the light transmitting in the core at the second ultra-abrupt taper. Both the length and diameter of the fabricated taper are only ∼100 μm, and the taper diameter-length ratio is around 1:1. Compared to the previously reported abrupt fiber tapers whose diameter-length ratios are around 1:10 [5,6], the taper fabricated by fusion-splicing is an *ultra-abrupt* one. It is more robust than MZIs consisting of stretched fiber tapers with the length of ∼600 μm and diameter of ∼60 μm [5,6].

**Figure 3.**
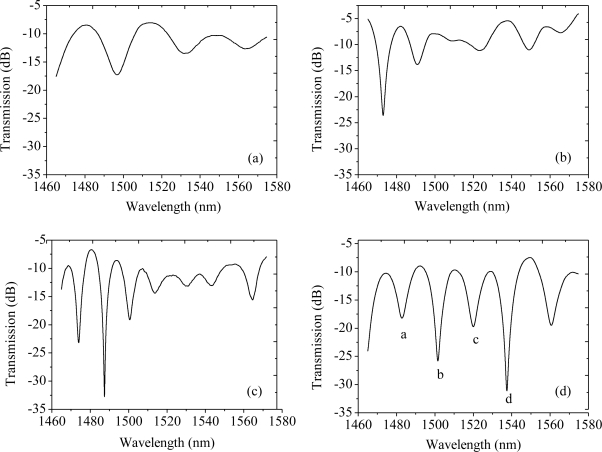
Transmission spectra of MZIs with different interferometer lengths: **(a)** 20 mm, **(b)** 30 mm, **(c)** 36 mm and **(d)** 40 mm.

A detection system consisting of a tunable laser (Agilent 81980A) and an optical power meter (Agilent 8163B) is employed to measure the transmission spectra by wavelength sweeping. The tunable laser scans through its wavelength range (1,465–1,575 nm) at the rate of 5 pm per step. The transmission spectra of fiber in-line MZI with different lengths are shown in Figure 3. Clear interference fringes are obtained with the maximum attenuations of 9 dB, 18 dB, 25 dB and 22 dB for interferometer length of 20 mm, 30 mm, 36 mm and 40 mm, respectively. The background loss is around 8 dB.

## 4. Sensor Applications

### 4.1. Measurements of Refractive Index Change

The effective refractive index of the cladding mode changes with the variations of external refractive index, and it results in a shift of the maximum attenuation wavelength. The sensitivity can be expressed as [7]:

(2) $$S\;=\;\frac{d\lambda_{v}}{dn_{\mathrm{ext}}}\;=\;-\frac{2L}{2k\;+\;1}\frac{\partial n_{\mathrm{cl},m}^{\mathrm{eff}}}{\partial n_{\mathrm{ext}}}/\left[1\;-\;\frac{2L}{2k\;+\;1}\left(\frac{\partial n_{\mathrm{core}}^{\mathrm{eff}}}{\partial \lambda }\;-\;\frac{\partial n_{\mathrm{cl},m}^{\mathrm{eff}}}{\partial \lambda }\right)\right]$$

where *n_ext_* is the external refractive index; *L* is the interferometer length; *k* is an integer; *λ_v_* is the maximum attenuation wavelength of the *k*th order; $n_{\mathrm{core}}^{\mathrm{eff}}$ and $n_{\mathrm{cl}}^{\mathrm{eff}}$ are the effective refractive indices of the core and cladding, respectively.

**Figure 4.**
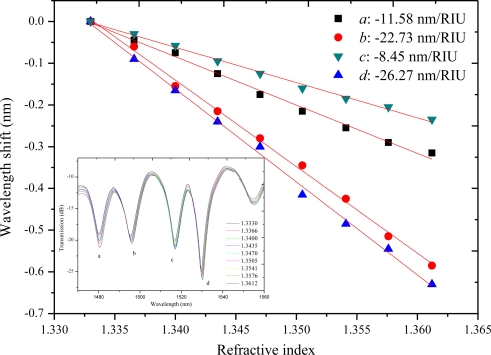
Wavelength shift of the four attenuation peaks due to external refractive index change, where *a*, *b*, *c* and *d* denote the various peaks. The inset shows the changes of the measured spectra.

The MZI with a length of 40 mm is tested as a refractive index sensor. Salt solutions with various concentrations (0.0%, 1.96%, 3.85%, 5.66%, 7.41%, 9.09%, 10.71%, 12.28%, 13.79%, mass percent) are used in the experiments. The corresponding RIs are 1.3330, 1.3366, 1.3400, 1.3435, 1.3470, 1.3505, 1.3541, 1.3576, and 1.3612, respectively [20]. Four attenuation peaks are chosen as the record wavelengths, which are marked by *a*, *b*, *c* and *d* in Figure 3(d). It denotes the maximum attenuation wavelengths of 1,486.92, 1,501.55, 1,519.85 and 1,537.56 nm, respectively. Figure 4 shows the shift of the four maximum attenuation wavelengths with the respect to the external RI change. All the attenuation peaks shift to shorter wavelength region with the increase of external refractive index. The sensitivities are −11.58, −22.73, −8.45 and −26.27 nm/RIU, respectively. In the RI range of 1.333–1.3648, the sensitivity is similar to those of LPFG pair MZI sensors [10] and the tapered-fiber pair MZI sensors [5,6]. It also demonstrates that refractive index sensitivity varies with the maximum attenuation peaks. The main reason is that different cladding modes have different mode field areas, and the sensitivities are different for various cladding modes. As a recent work reported by Li *et al.* [21], higher order cladding modes have lager mode field areas and are more easily affected by the surrounding refractive index, hence, higher order cladding modes generally exhibit lower mode indices and higher sensitivities. The different sensitivity results showed in our study are in accordance with the peak shift discrepancy reported in [21].

### 4.2. Temperature Measurement

**Figure 5.**
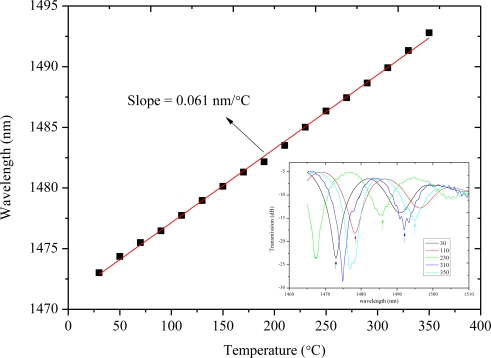
Temperature response of the proposed MZI at a length of 30 mm. The inset shows the changes of the measured spectra.

Due to the difference between the effective refractive index changes of cores and those of claddings in response to the temperature variations, the MZI can be used as a temperature sensor. In a muffle furnace, a 30 mm long MZI is used to test temperature sensitivity. The temperature changes every 5 min from 30 °C to 350 °C with a step of 20 °C. Figure 5 shows the attenuation maximum wavelength (around 1,473 nm) shift as a function of the temperature where the relation is roughly linear. A red shift occurs as the temperature increases, and the sensitivity is 0.061 nm/°C through a linear-fitting of the measured data, which is as good as those of grating-based temperature sensors [22]. The sensitivity of temperature is about 0.02 °C with the detection system of 1 pm resolution.

### 4.3. Angular Displacement Measurement

**Figure 6.**
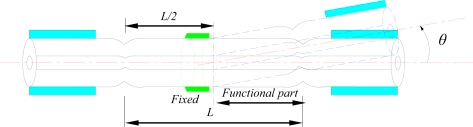
The structural diagram of the proposed MZI used as angular-displacement sensor.

In this paper, the proposed MZI is used to measure angular displacement by fixing it on a micro-rotation stage. The corresponding structural diagram is shown in Figure 6.

**Figure 7.**
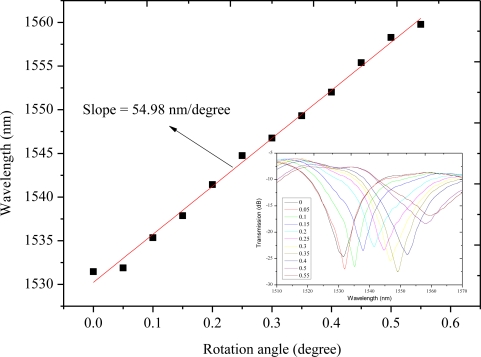
The attenuation maximum wavelength shift due to the changes of angular displacement. The inset shows the changes of the measured spectra.

Because of the macro-bending, the coupling of the core mode to the cladding modes is changed by the variation of angle *θ*. By recording the wavelength shift in the transmission spectrum, we can test the angular displacement. An MZI with length of 25 mm is chosen to test the angular-displacement sensitivity in the angle range from 0° to 0.55° with an increment of 0.05° for a total of 11 steps. Figure 7 shows the attenuation maximum wavelength (around 1,530 nm) shift as a function of the angle *θ*. The sensitivity is 54.98 nm/° with good linearity. At a resolution of 1pm of the detecting system, the detection limit is 3.17 × 10^−7^ rad. Compared to other methods [23], the proposed MZI sensors are of many advantages including structure simpleness, reliability, compactness, robustness, low detection limit, and simple low-cost fabrication process.

### 4.4. RI Sensitivity Improved by the Femtosecond Laser

**Figure 8.**
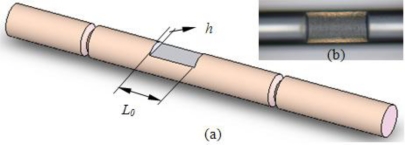
Micro-trench fabricated by femtosecond laser pulses. **(a)** Schematic diagram, **(b)** Top view of the micro-trench with *h* = 18 μm and *L_0_* = 200 μm.

In order to improve the RI sensitivity of the proposed MZI sensor, an inline side-ablated micro-trench was fabricated to remove part of the fiber cladding using a femtosecond laser. The micro-trench lies in the center of the MZI, as shown in Figure 8(a). When part of the fiber cladding is removed, the optical power related to the evanescent wave of cladding modes significantly increases in the external medium. Thus, the enhancement of sensitivity is expected when the fiber cladding of the interaction area between the sensor and the medium is reduced.

**Figure 9.**
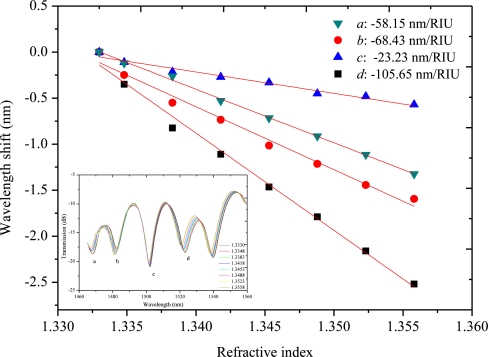
For the MZI imbedded with a micro-trench, wavelength shift of the four attenuation peaks (*a*, *b*, *c* and *d*) due to external RI changes. The inset is the measured transmission spectra.

A side-ablated micro-trench is fabricated in the aforementioned MZI (*L* = 40 mm) to improve the RI sensitivity. Figure 8(b) shows microscopic image of the fabricated micro-trench with an ablated depth (*h*) of 18 μm and a length (*L_0_*) of 200 μm, respectively. The width of 88 μm is obtained accordingly for the fiber is cylinder. The measured salt solutions are the same as in session 4.1. Salt solutions with various concentrations (0.0%, 0.99%, 2.91%, 4.76%, 6.54%, 8.26%, 9.91%, 11.50%, mass percent) are used in the experiments. The corresponding RIs are 1.3330, 1.3348, 1.3383, 1.3418, 1.3453, 1.3488, 1.3523 and 1.3558, respectively [20]. For the MZI imbedded by a micro-trench, the attenuation peaks marked by *a*, *b*, *c* and *d* in Figure 3(d) are changed to 1,483.4, 1,501.86, 1,520.72 and 1,538.06 nm, respectively. Figure 9 shows the shift of the four maximum attenuation wavelengths with respect to the external RI change. All the attenuation peaks shift to shorter wavelength region with the increase of external refractive index. The improved sensitivity becomes −58.15, −68.43, −23.23 and −105.65 nm/RIU, respectively. The sensitivity is improved by 3–5 fold for various interference modes. This value is comparable to LPFG sensors after specially designed for sensitivity enhancement [24].

### 4.5. Zeolite Coated MZI Sensor for Organic Vapors Tests

Zeolites are aluminosilicate materials with three dimensional crystalline porous skeletons, possessing unique chemical and optical properties which can be used to develop highly sensitive optical chemical sensors. The large surface-to-mass ratio of these pores could also efficiently adsorb (thus collect and concentrate) molecules from the ambient for highly sensitive detection. In this paper, MFI-type zeolite thin film is synthesized on the MZI for detection of acetone vapors with high sensitivity. The zeolite film is coated on the MZI cylindrical surface by *in situ* crystallization from an aluminum-free precursor solution using tetrapropylammonium ion as the structure directing agent. The details for zeolite coating are given in [14].

**Figure 10.**
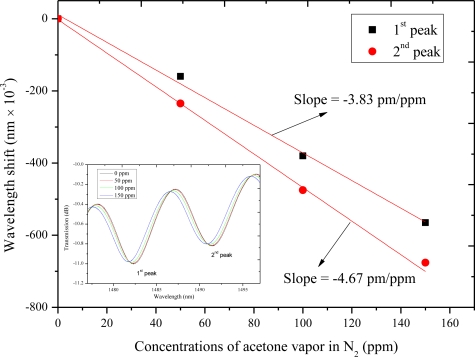
The shift of the two attenuation peaks due to the changes of acetone vapor concentrations. The inset is the measured transmission spectra of the MZI sensor response to various concentrations of acetone vapor in N_2_.

The sensor measure gas concentrations by monitoring the molecular adsorption-induced changes of the interference pattern position. The sensitivity is affected by many parameters such as the type of the zeolite, the film thickness, the measurands and the characteristics of the MZI. The length of the MZI is 36 mm, while the transmission spectrum is changed completely after being coated with zeolite film (as shown in Figure 10). We study the sensitivities of two maximum attenuation peaks (marked in Figure 10) in the transmission spectra. As the acetone vapor concentration increases, a blue shift is observed in the transmission spectra. The sensitivities of the 1st and 2nd attenuation peaks are −3.83 pm/ppm and −4.67 pm/ppm through linear fitting, respectively.

## 5. Conclusions

High-quality fiber inline MZIs are fabricated by a new fusion-splicing method using ordinary single mode fibers. By fusion-splicing, the taper diameter-length ratio is 1:1, which is much greater than those (1:10) achieved by stretching. The fabricated MZIs are applied to sense refractive index, temperature and rotation angle changes. The temperature sensitivity is 0.061 nm/°C in 30–350 °C. At a resolution of 1 pm of the detecting system, a detection limit is 3.17 × 10^−7^rad when the proposed MZI is used to measure angular displacements. The proposed fabrication method is very low cost, 1/20–1/50 that of LPFG pair MZI sensors. The RI sensitivity is improved by 3–5 fold after an inline side micro-trench is ablated by a femtosecond laser on the fiber cladding. In addition, the MZI sensor is used to measure acetone vapor of 50 ppm in N_2_ after being coated with zeolite thin film. The MZI sensors are of very high fabrication and sensing repeatability, thus offering many advantages including structure simpleness, reliability, compactness, robustness, high sensitivity, high flexibility and simple low-cost fabrication process. Moreover, such a robust and low-cost MZI sensor is capable of *in situ* detection in many areas such as environmental management, industrial process control, and public health.
